# Supplementary material for: Bioactive Nanofiber-Based Conduits in a Peripheral Nerve Gap Management—An Animal Model Study
Source: Int J Mol Sci. 2021 May 25;22(11):5588. doi: 10.3390/ijms22115588 (PMC8197537; doi:10.3390/ijms22115588)
Supplement: Supplementary file 1 [file ijms-22-05588-s001.zip › ijms-1215335-supplementary.pdf]

Supplementary Material to:

Bioactive nanofiber-based conduits in a peripheral nerve gap management- an animal model study.

Tomasz Dębski [1,*], Ewa Kijeńska-Gawrońska [2,3], Aleksandra Zołocińska [1], Katarzyna Siennicka [1], Anna Słysz [1], Wiktor Paskal [4], Paweł K. Włodarski [4], Wojciech Święszkowski [3] and Zygmunt Pojda [1]

[1] Department of Regenerative Medicine, Maria Sklodowska-Curie National Research Institute of Oncology, Wawelska 15B, 02-034 Warsaw,
[2] Centre for Advanced Materials and Technologies CEZAMAT, Warsaw University of Technology, Poleczki 19, 02-822 Warsaw, Poland
[3] Materials Design Division, Faculty of Materials Science and Engineering, Warsaw University of Technology, Woloska 141, 02-507 Warsaw, Poland
[4] Department of Methodology, Centre for Preclinical Research, Medical University of Warsaw, Banacha 1b, 02-097 Warsaw, Poland

Contents:

1. SEM images of control P(LLA-CL)-PANI fibrous mats used for the cell *in vitro* studies. (Figure S1)

2. A table for physio-chemical results obtained for control P(LLA-CL)-PANI fibrous mats used for the cell *in vitro* studies. (Table S1)

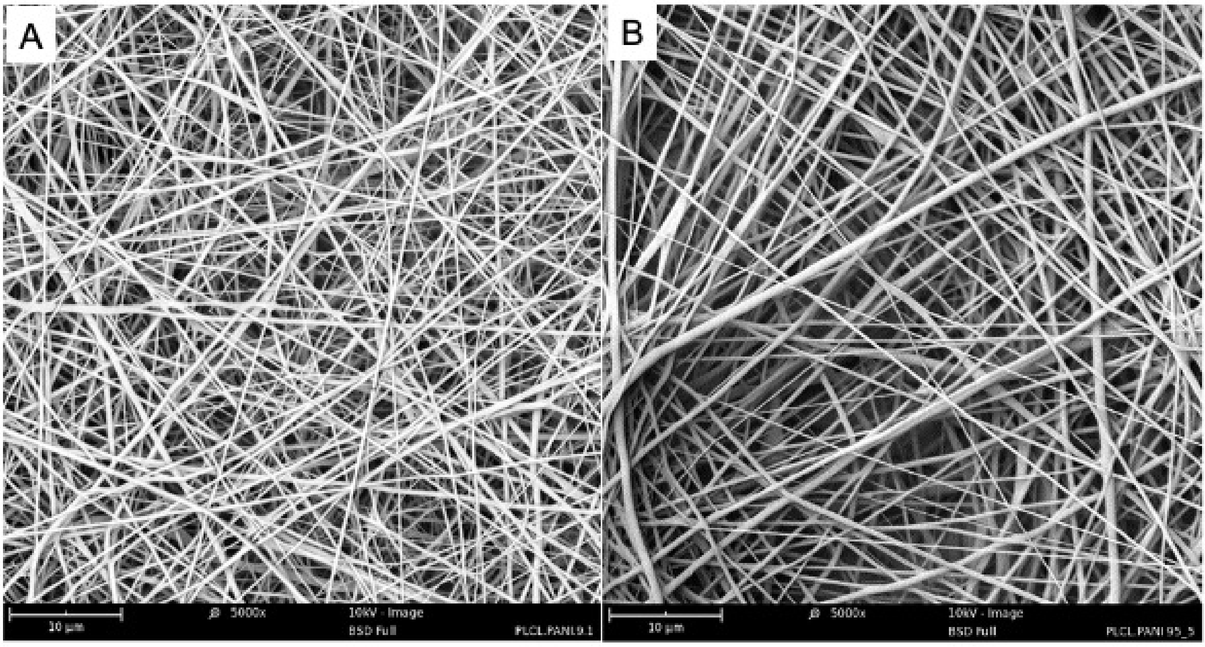

Figure S1. SEM images of control P(LLA-CL)-PANI fibrous mats. (A) Magnification x2,5; (B) Magnification x5000

Table S1. Parameters of P(LLA-CL)- PANI mats

Parameter	Value	Units
Mean fiber thickness	377 ± 162	nm
Tensile strength	3.31± 1.01	MPa
Elongation to break	137.97 ± 32.554	%
Contact angle	120.05 ± 9.30	º
